# Partial tumor irradiation plus pembrolizumab in treating large advanced solid tumor metastases

**DOI:** 10.1172/JCI162260

**Published:** 2023-05-15

**Authors:** Mark C. Korpics, Benjamin E. Onderdonk, Rebekah E. Dadey, Jared H. Hara, Lilit Karapetyan, Yuanyuan Zha, Theodore G. Karrison, Adam C. Olson, Gini F. Fleming, Ralph R. Weichselbaum, Riyue Bao, Steven J. Chmura, Jason J. Luke

**Affiliations:** 1 Department of Radiation and Cellular Oncology, The University of Chicago, Chicago, Illinois, USA.; 2 UPMC Hillman Cancer Center and University of Pittsburgh School of Medicine, Pittsburgh, Pennsylvania, USA.; 3 Department of Cutaneous Oncology, H. Lee Moffitt Cancer Center and Research Institute, Tampa, Florida, USA.; 4 Human Immunological Monitoring Core, Biological Sciences Division,; 5 Department of Public Health Sciences,; 6 Department of Medicine, Section of Hematology/Oncology, and; 7 Ludwig Center for Metastasis Research, The University of Chicago, Chicago, Illinois, USA.

**Keywords:** Clinical Trials, Oncology, Cancer, Immunotherapy, Radiation therapy

## Abstract

**BACKGROUND:**

We previously demonstrated the safety of stereotactic body radiotherapy followed by pembrolizumab (SBRT+P) in patients with advanced solid tumors. This phase I clinical trial was expanded to study the safety of partial tumor irradiation (partial-Rx). We assessed irradiated local failure (LF) and clinical outcomes with correlations to biomarkers including CD8^+^ T cell radiomics score (RS) and circulating cytokines.

**METHODS:**

Patients received SBRT to 2–4 metastases and pembrolizumab for up to 7 days after SBRT. Tumors measuring up to 65 cc received the full radiation dose (complete-Rx), whereas tumors measuring more than 65 cc received partial-Rx. Landmark analysis was used to assess the relationship between tumor response and overall survival (OS). Multivariable analysis was performed for RS and circulating cytokines.

**RESULTS:**

In the combined (expansion plus original) cohort, 97 patients (219 metastases) were analyzed and received SBRT+P. Forty-six (47%) patients received at least 1 partial-Rx treatment. There were 7 (7.2%)dose-limiting toxicities (DLTs). 1-year LF was 7.6% overall, and 13.3% and 5.4% for partial-Rx and complete-Rx tumors, respectively (HR 2.32, 95% CI 0.90–5.97, *P* = 0.08). The overall, unirradiated, and irradiated objective response rates were 22%, 12%, and 34%, respectively. Irradiated tumor response to SBRT+P was associated with prolonged OS; 1-year OS was 71% (responders), 42% (mixed-responders), and 0% (nonresponders) (*P* < 0.01). High-RS was significantly associated with improved LF, progression-free survival (PFS), and OS. Elevated circulating IL-8 was independently associated with inferior PFS and OS.

**CONCLUSION:**

SBRT+P is safe in patients with large, advanced solid tumors. Additional studies are warranted to assess noninferiority of complete versus partial irradiation of tumors in the setting of immunotherapy.

**TRIAL REGISTRATION:**

Clinicaltrials.gov NCT02608385

**FUNDING:**

Merck Investigator Studies Program; Hillman Fellows for Innovative Cancer Research Program; NIH grants UM1CA186690-06, P50CA254865-01A1, P30CA047904-32, and R01DE031729-01A1.

## Introduction

Immune-checkpoint inhibitor immunotherapy and stereotactic body radiotherapy (SBRT) are powerful stand-alone cancer therapies and mechanistic studies support combination regimens (1). Numerous potential mechanisms of combined SBRT and immunotherapy efficacy have been postulated, including increased tumor antigen exposure, improved antigen presentation by dendritic cells or T cell function, modulation of immunosuppressive cell populations such as T regulatory cells or myeloid derived suppressor cells, and cytoreduction of large immune-excluded tumors (1–7). The combination of SBRT with immunotherapy has numerous potential advantages, and combination approaches are under intense investigation. Despite the excitement surrounding combination SBRT and immunotherapy, prospective studies investigating this treatment regimen have produced mixed results (8, 9).

Historically, SBRT for large tumors, tumors measuring more than 5 cm or more than 65 cc, was considered neither safe nor feasible due to nearby critical organs at risk (OARs) and/or radiation dose tolerance, which is based on the probability of normal tissue complications (10–13). This constraint has led to a conditional recommendation for SBRT use in early stage lung cancer by the American Society for Radiation Oncology (14). Based on these recommendations, a maximum size of 5 cm (65 cc) remains an acceptable inclusion criterion for SBRT in radiation oncology clinical trials (15, 16). Similarly, trials investigating SBRT with pembrolizumab (SBRT+P) in recurrent metastatic lung cancer have used a 5cm maximum size inclusion criterion (17). Whether SBRT combined with immunotherapy is feasible or efficacious in patients with large, advanced, solid tumors remains unknown.

We previously demonstrated that the combination of multi-site SBRT+P (18) was well tolerated in patients with advanced solid tumors. In that report, 18 of 68 patients (21 large metastases) received a prescription dose of radiation to a limited portion of their metastatic tumors (termed partial-Rx). The intent was to limit toxicity and to assess the preliminary therapeutic outcomes of this approach. On exploratory analysis, these large tumors had similar local control to tumors treated completely (complete-Rx; *P* = 0.24) (19). However, the small sample size and short survival follow up limited the interpretation of these results.

There is a paucity of clinically useful biomarkers to identify patients that may benefit from combined SBRT and immunotherapy. In our initial reported cohort, we investigated several clinical and molecular biomarkers and found that response of the irradiated metastasis (19) was associated with reduced risk of death. In addition, we observed gene expression changes from before and after SBRT biopsies, suggesting that tumor microenvironment was modulated by high-dose SBRT toward increased expression of innate and adaptive immune genes and reduced expression of DNA repair and cell cycle genes (19). In particular, tumors that demonstrated increased expression of cytolytic T cell genes after SBRT had an improved tumor response.

Noninvasive means to evaluate immune infiltration of tumors and predict response are also of interest. A validated CD8^+^ T cell radiomics score (RS), generated using 5 radiomic features extracted from conventional CT images, has demonstrated utility in predicting CD8^+^ T cell infiltration in patients receiving immunotherapy (20). In the context of radiation and immunotherapy, the RS may be used to predict survival and response outside of the unirradiated metastases (21). We previously investigated the role of the pretreatment T cell RS in patients who received SBRT+P and found that a high RS was associated with improved progression-free survival (PFS) and overall survival (OS) (22), suggesting the utility of RS in this setting.

Circulating cytokines, notably IL-8, have also been associated with outcome to PD1/L1 checkpoint blockade (23–25). Elevated serum IL-8 is associated with increased intratumoral immuno-suppressive neutrophils, despite high CD8^+^ T cell infiltration and lack of response in melanoma, renal cell carcinoma, non-small cell lung carcinoma, and urothelial carcinoma. We similarly observed an association of elevated IL-8 with lack of treatment response in a study of SBRT with combination immunotherapy (26). Together, our prior work suggests that SBRT+P is safe and feasible, and that factors beyond tumor size or complete tumor irradiation, such as RS, expression of IFN-related pathways, and the presence of certain circulating cytokines, may predict outcomes.

Here, we describe the safety and preliminary efficacy of SBRT+P in a dedicated cohort of patients with advanced solid tumors with the incorporation of an expansion cohort of patients with large, partially irradiated tumors. We explore partial-Rx and complete-Rx tumor control, the implications of tumor response, RS, and whether cytokines associated with innate and adaptive immune pathways can be prognostic for clinical outcomes (18, 19, 22).

## Results

### Patient characteristics and toxicity in the large tumor expansion cohort.

Since the original publication, 41 additional patients with at least 1 large (more than 65 cc) metastasis were enrolled into an expansion cohort with SBRT administered to 117 metastases. Baseline characteristics are listed in Table 1. Colorectal cancer was the most common primary cancer (32%). Sixteen patients received SBRT to 2 metastases, 15 received SBRT to 3 metastases, and 10 received SBRT to 4 metastases. Each patient had at least 1 metastasis that received partial-Rx. Of the 117 metastases, 66 metastases (56.4%) were more than 65cc and received partial-Rx and 51 metastases (43.6%) received complete-Rx. Of those 41 patients enrolled who started SBRT, 2 did not complete SBRT, 4 completed SBRT but did not receive pembrolizumab, and 7 completed SBRT+P but did not receive imaging follow up. Of the 41 patients in the expansion cohort, 28 patients were considered analyzable — they completed SBRT, received at least 1 cycle of pembrolizumab, and were evaluable for toxicity and tumor control. The 1-year cumulative incidence of irradiated local failure (LF), accounting for the competing risk of death, was 5.3%. The Response Evaluation Criteria in Solid Tumors (RECIST) v1.1, modified to include irradiated tumors as target lesions, best objective response rate was 21%. The unirradiated and irradiated ORR were 13% and 25%, respectively.

Among those available for analysis, there was only 1 dose-limiting toxicity (DLT) in the expansion cohort, grade 3 pneumonitis, which occurred in a patient with uterine leiomyosarcoma who received 3 cycles of pembrolizumab and SBRT to 3 central lung metastases. The patient had a past medical history of asthma with exacerbations requiring steroids in the prior 4 months, was a former smoker, had no prior radiation, and previously received 3 lines of systemic therapy (no immunotherapy). The patient demonstrated a mixed response to SBRT+P and was admitted to the intensive care unit for type 2 respiratory failure approximately 6 weeks after completion of SBRT and 1 week after her third cycle of pembrolizumab. All lung dose constraints per protocol were met.

### Patient characteristics and toxicity of the combined cohorts.

In the combined cohort, which included the large tumor expansion cohort plus the original study cohort, 117 patients with 275 metastases were enrolled and started SBRT. Baseline characteristics are shown in Table 1. Head and neck cancer was the most common primary for complete-Rx patients, and colorectal cancer was the most common primary for partial-Rx patients. Eighty-seven patients received SBRT to 2 metastases, 19 patients received SBRT to 3 metastases, and 11 patients received SBRT to 4 metastases. Of those 117 patients that initially enrolled and started SBRT, 3 did not complete SBRT, 6 completed SBRT but did not receive pembrolizumab, and 11 completed SBRT+P but did not receive imaging follow up. Of the 117 patients in the combined cohort, 97 patients with 219 metastases were considered analyzable. There were 46 patients (47.4%) with at least 1 metastasis that received partial-Rx and 51 patients (52.6%) that received complete-Rx to all metastases. On a tumor level, 62 metastases (28.3%) received partial-Rx and 157 metastases (71.7%) received complete-Rx. The median tumor size and ranges for those that received all complete-Rx and those with at least 1 partial-Rx were 8.4 cc (0.4–63.4 cc) compared with 137.9 cc (49.8–2720.5 cc) (*P* < 0.01), respectively.

There was a total of 7 DLTs for an overall rate of 7.2%. All DLTs occurred in patients who were considered analyzable. 6 DLTs occurred in those that received only complete-Rx (6 of 51, 11.8%) and 1 DLT (1 of 46, 2.2%) in a patient that received partial-Rx (*P* = 0.03). The DLTs most frequent were 4 cases of grade-3 pneumonitis, followed by 2 cases of grade-3 colitis, and 1 case of grade-3 hepatic toxicity.

### Treatment response and association with survival in the combined cohorts.

**Figure 1:**
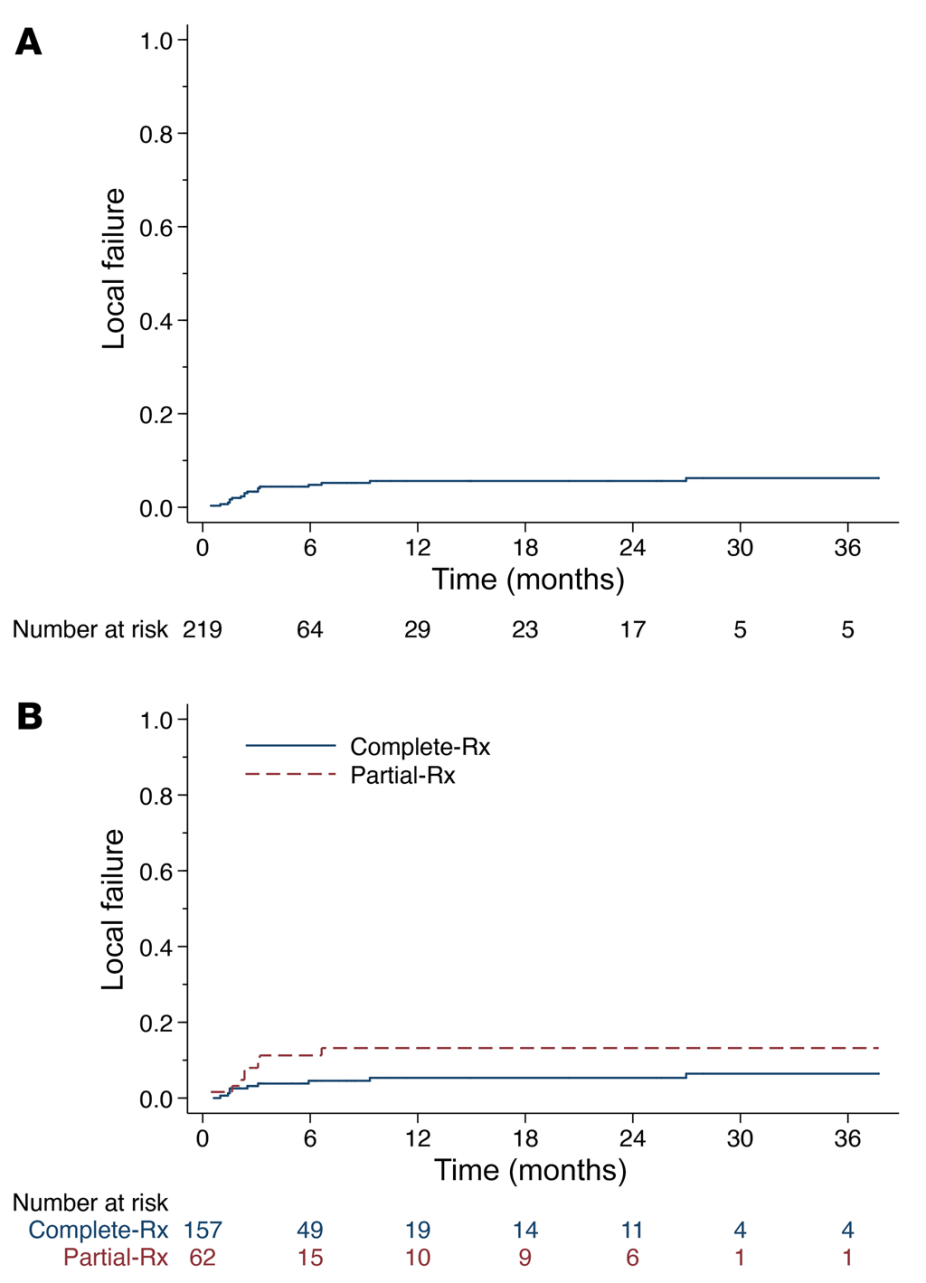
Irradiated LF. (**A**) Cumulative incidence of irradiated LF accounting for the competing risk of death (*n* = 219). (**B**) LF by prescription stereotactic body radiation therapy dose covering the entire metastasis (complete-Rx, *n* = 157) compared with partial metastasis coverage (partial-Rx, *n* = 62) (overall, *n* = 219). On competing risk regression accounting for the competing risk of death, tumors receiving partial-Rx did not have a significantly higher LF compared to tumors receiving complete-Rx (HR 2.32, 95% CI 0.90–5.97, *P* = 0.08).

**Figure 2:**
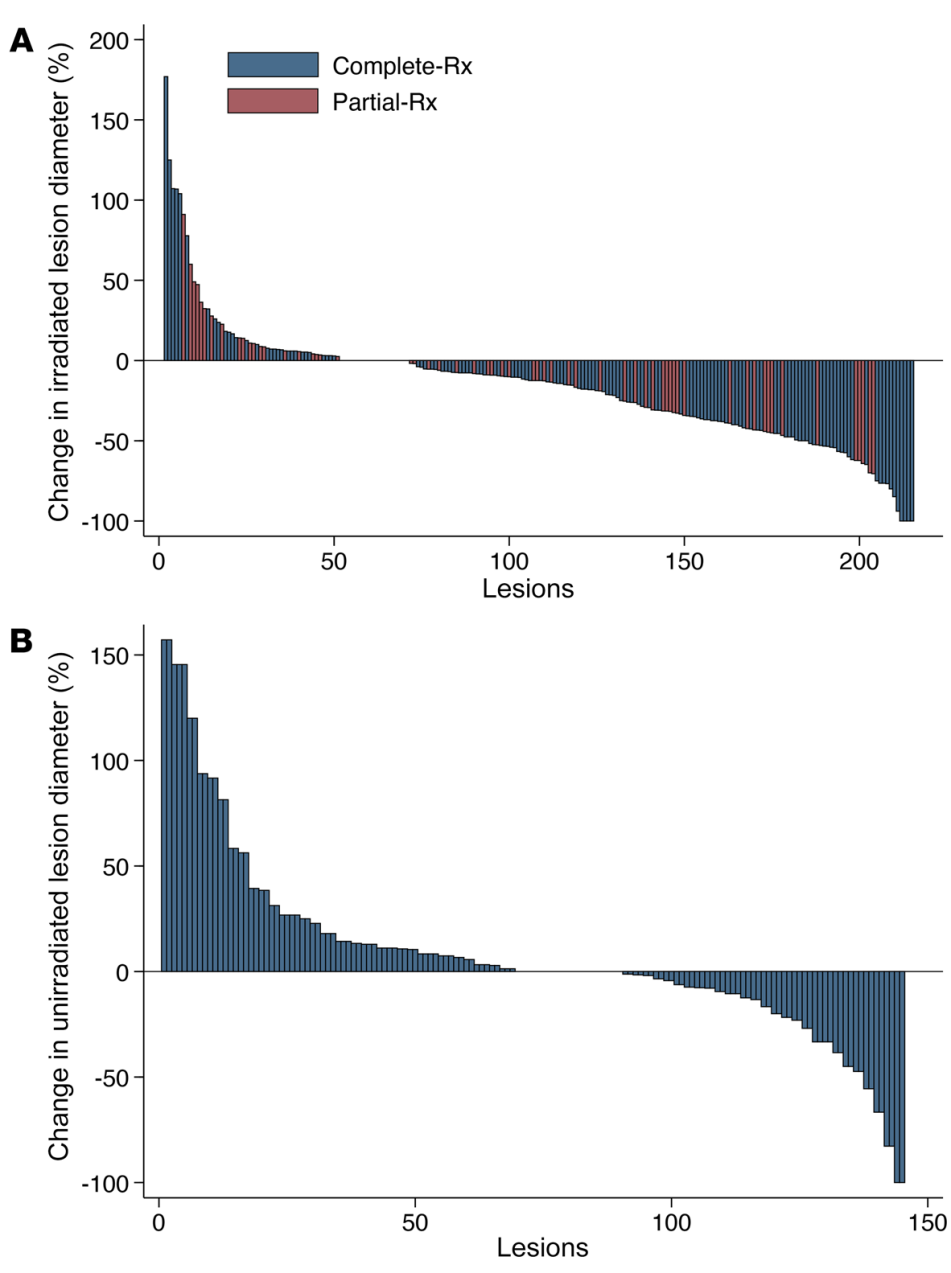
Metastasis response waterfall plots. (**A**) Change in irradiated lesion diameter stratified by prescription SBRT dose covering the entire metastasis (complete-Rx, *n* = 157) and partial metastasis (partial-Rx, *n* = 62) (overall, *n* = 219). One complete-Rx metastasis demonstrated a 947% increase in diameter and was excluded from the waterfall plot to improve visualization of the other metastasis responses. (**B**) Change in unirradiated lesion diameter (*n* = 145).

Similar to the analysis of the initial dose–determination cohort (19), the current analysis demonstrated a 7.6% 1-year cumulative incidence of LF, accounting for the competing risk of death (Figure 1A; per metastasis analysis). On competing risk regression, there was no statistically significant difference in LF for tumors receiving partial-Rx versus complete-Rx (Figure 1B; HR 2.32, 95% CI 0.90–5.97, *P* = 0.08); however, this study was not specifically designed to establish noninferior LF for tumors receiving partial-Rx versus complete-Rx. Additional analysis to account for the anatomical location of the tumor was conducted because 71% of LF events occurred in the liver. In addition, 36% of partial-Rx tumors involved liver tumors, whereas 18% of complete-Rx tumors were hepatic. Competing risk regression comparing partial-Rx and complete-Rx tumors, controlling for anatomical location, demonstrated a HR of 1.50 (95% CI 0.57–3.92, *P* = 0.41). The RECIST objective response rate was 22%. The unirradiated and irradiated ORR were 12% and 34%, respectively. A waterfall plot depicting response rate in complete-Rx, partial-Rx, and unirradiated tumors is shown in Figure 2. For patients receiving partial-Rx, LF did not differ based on PD-L1 presence/absence (*P* = 0.93) or tumor size quartile (*P* = 0.98). Supplemental Table 1 (supplemental material available online with this article; https://doi.org/10.1172/JCI162260DS1) shows the association of LF and select covariates, of which only anatomical site and RS were found to be associated with LF, while PD-L1 expression, histology, tumor volume, and other covariates were not associated with LF. On a per-patient basis, the median PFS was 3.2 months (95% CI 3.0–5.2) and the median OS was 9.1 months (95% CI 6.4–12.4). Of the patients not considered analyzable, the median PFS was 2.2 months (95% CI 1.0–4.5) and the median OS was 2.2 months (95% CI 0.9–4.5).

**Figure 3:**
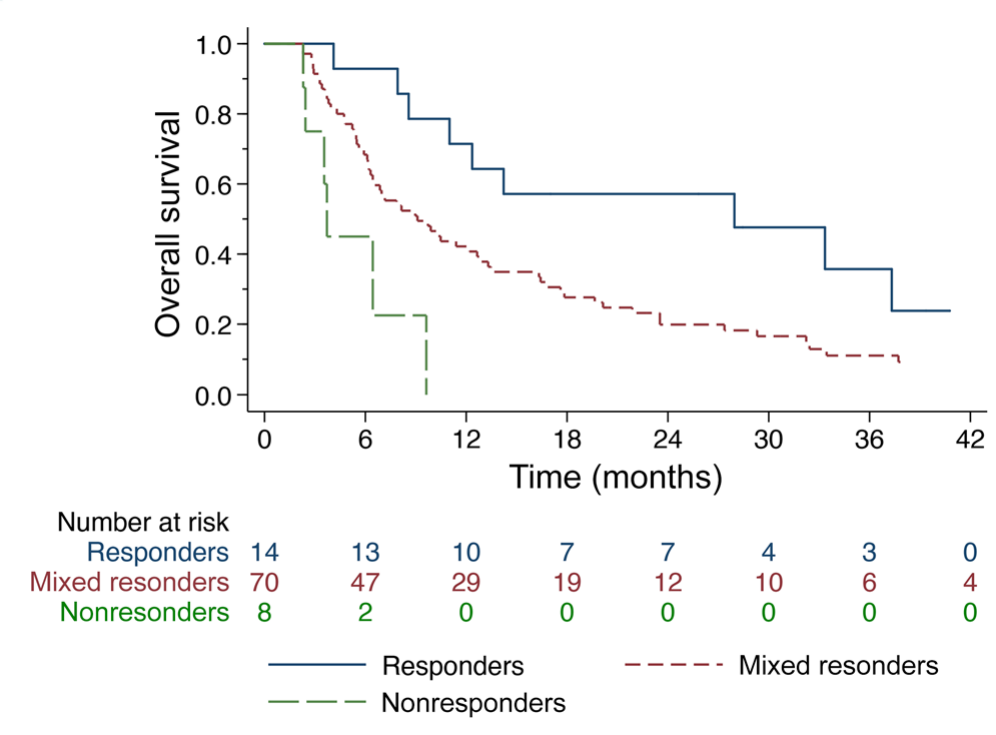
OS stratified by irradiated tumor response. OS stratified by irradiated tumor response (responders, mixed responders, and nonresponders) from those that survived to the 2-month landmark time (*n* = 92). There were 14 responders, 70 mixed-responders, and 8 nonresponders. Overall Log-rank *P* <0.01. On Cox PH model, the HRs were: responders versus mixed-responders (0.46, 95% CI 0.23–0.93, *P* = 0.03), responders versus non-responders (0.14, 95% CI 0.05–0.40, *P* < 0.01), and mixed-responders versus nonresponders (0.30, 95% CI 0.12–0.72, *P* < 0.01).

Of the 92/117 (78.6%) patients who survived to the 2-month landmark time, 14 were responders (all irradiated tumors responded), 8 were nonresponders (at least 1 irradiated tumor progressed), and the remaining 70 patients had a mixed response (not all irradiated tumors responded but none progressed). The 1-year OS was 71% for responders, 42% for mixed-responders, and 0% for nonresponders (Figure 3; *P* < 0.01). The HRs for each group were as follows: responders versus mixed-responders (0.46, 95% CI 0.23–0.93, *P* = 0.03), responders versus nonresponders (0.14, 95% CI 0.05–0.40, *P* < 0.01), and mixed-responders versus nonresponders (0.30, 95% CI 0.12–0.72, *P* < 0.01). A multivariable Cox proportional hazards (PH) model controlling for serum albumin, the number of prior systemic therapies, age, and performance status, demonstrated that irradiated metastasis response was independently associated with OS (Table 2).

### Radiation dosimetry in the combined cohorts.

To better characterize the tumor coverage by the prescription dose, additional dosimetric comparisons were made between partial-Rx and complete-Rx tumors. The planning target volume (PTV) is the target of the prescription dose and is commonly defined as the tumor plus margin to allow for setup uncertainty. Coverage of the PTV by the radiation prescription dose was assessed using 2 metrics: the percentage of the prescription dose received by 100% of the PTV (D100%) and the percentage of the prescription dose received by the hottest 90% of the PTV (D90%). The study protocol required that sparing of OARs be prioritized over tumor coverage. Therefore, the D100% and D90% in this patient cohort are lower than what are commonly seen in conventional SBRT plans. For complete-Rx versus partial-Rx tumors, the median (interquartile range) D100% was 75% (52%–90%) and 49% (18%–82%), respectively, and the median D90% was 99% (85%–102%) and 82% (46%–99%), respectively. Comparing complete-Rx versus partial-Rx tumors, both the D100% (*P* < 0.001) and D90% (*P* < 0.001) were significantly different, which indicates a significantly lower dose to the entire tumors in partial-Rx compared with complete-Rx tumors. Taking these differences in tumor coverage into consideration, neither the D100% (*P* = 0.54) nor D90% (*P* = 0.27) were associated with LF.

### Within-patient comparison of tumor response by tumor coverage.

There were 37 patients that received a combination of both complete-Rx (for tumors of at least 65 cc) and partial-Rx (for tumors more than 65 cc) treatments, thus, 2 categories of response were defined. Of these 37 patients, 84% (31 patients) had a convergent response, which was defined as either response or stability of both complete-Rx and partial-Rx tumors. The remaining 16% (6 patients), demonstrated a divergent response, which was defined as irradiated tumors that do not meet the convergent criteria (e.g., a partial-Rx tumor progressed while a complete-Rx tumor responded or remained stable). Of note, 1 of the 6 patients with a divergent response had a partial-Rx tumor respond while a complete-Rx tumor progressed.

### RSs in the combined cohorts.

**Figure 4:**
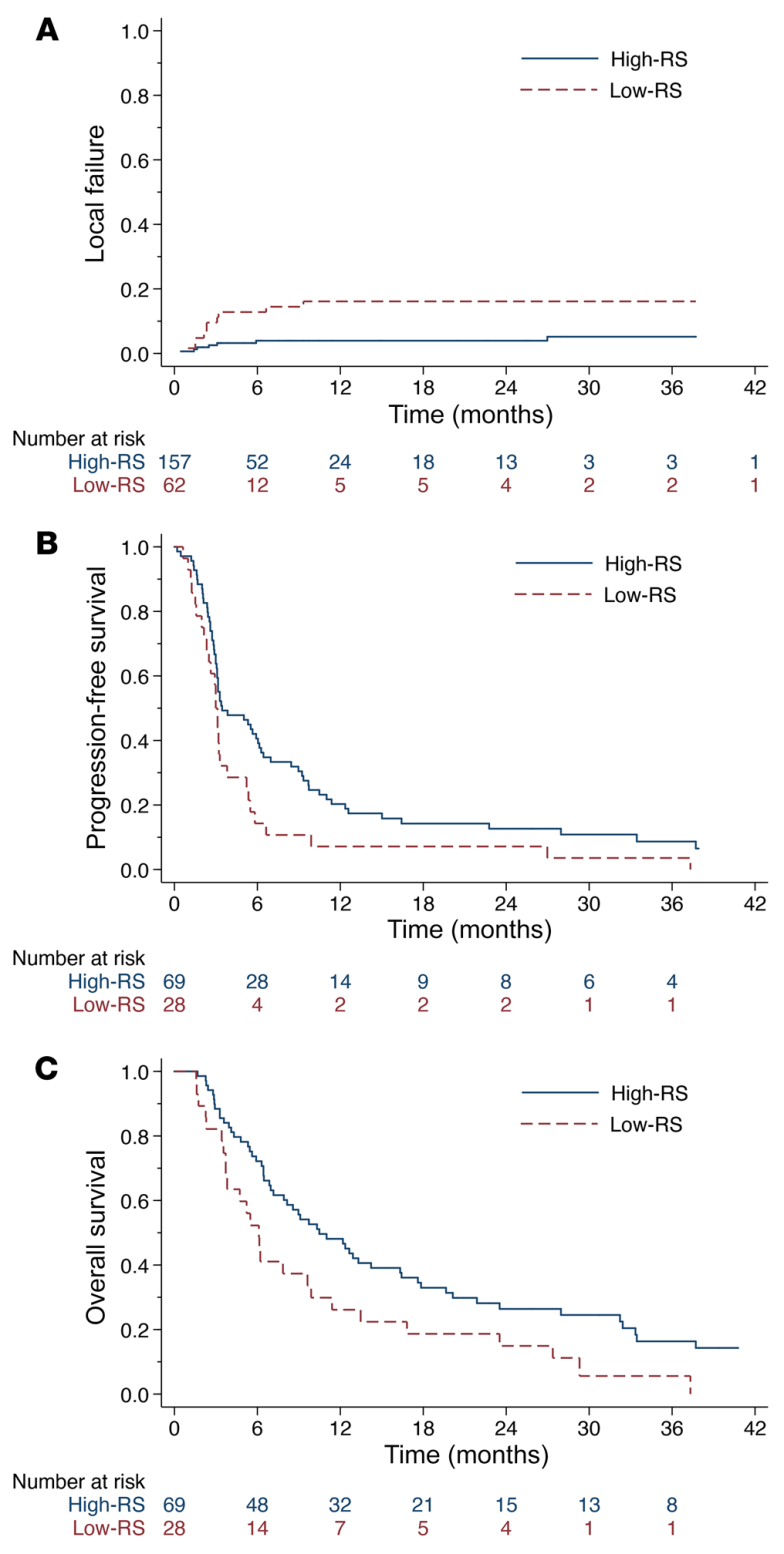
Clinical outcomes stratified by RS. (**A**) Cumulative incidence of irradiated LF accounting for the competing risk of death stratified by prespecified metastasis-level RS cutoff of 0.42. On competing risk regression accounting for the competing risk of death, the HR for high-RS versus low-RS was 0.27 (95% CI 0.10–0.70, *P* < 0.01). (**B**) Kaplan-Meier estimates of PFS stratified by prespecified patient-level RS cutoff of 0.53. Metastases in the high-RS group (*n* = 69) were associated with improved PFS compared with the low-RS group (*n* = 28). Log-rank *P* = 0.02. On Cox PH model, the HR for high-RS versus low-RS was 0.59 (95% CI 0.37–0.93, *P* = 0.02). (**C**) Kaplan-Meier estimates of OS stratified by prespecified patient-level RS cutoff of 0.53. Metastases in the high-RS group (*n* = 69) were associated with improved OS compared with the low-RS group (*n* = 28). Log-rank *P* = 0.01. On Cox PH model, the HR for high-RS versus low-RS was 0.56 (95% CI 0.35–0.90, *P* = 0.01).

The metastasis-level RS, as a continuous variable, was not associated with PD-L1 positivity (*P* = 0.22) or PD-L1 percentage (*P* = 0.40). There was a significant difference in metastasis-level RS when comparing tumors that responded versus tumors that progressed (*P* = 0.02). When comparing the metastasis-level RS for partial-Rx versus complete-Rx tumors, there was no significant difference (*P* = 0.35). Using the previously reported low- and high-RS cutoff values at the metastasis-level (22), high-RS was associated with improved LF (Figure 4A; competing-risks regression HR 0.27, 95% CI 0.10–0.70, *P* < 0.01); which remained significant (HR 0.29, 95% CI 0.11–0.77, *P* = 0.01) when controlling for tumor coverage (partial-Rx versus complete-Rx: HR 2.00, 95% CI 0.77–5.21, *P* = 0.16). At the patient level, patients in the high-RS group had improved PFS (Figure 4B; HR 0.59, 95% CI 0.37–0.93, *P* = 0.02), and improved OS (Figure 4C; HR 0.56, 95% CI 0.35–0.90, *P* = 0.02). The RS, as a continuous variable, was significantly associated with LF (*P* < 0.01) and PFS (*P* = 0.04), but it was not associated with OS (*P* = 0.15).

### IL-8 levels correlate with inferior OS and PFS.

**Figure 5:**
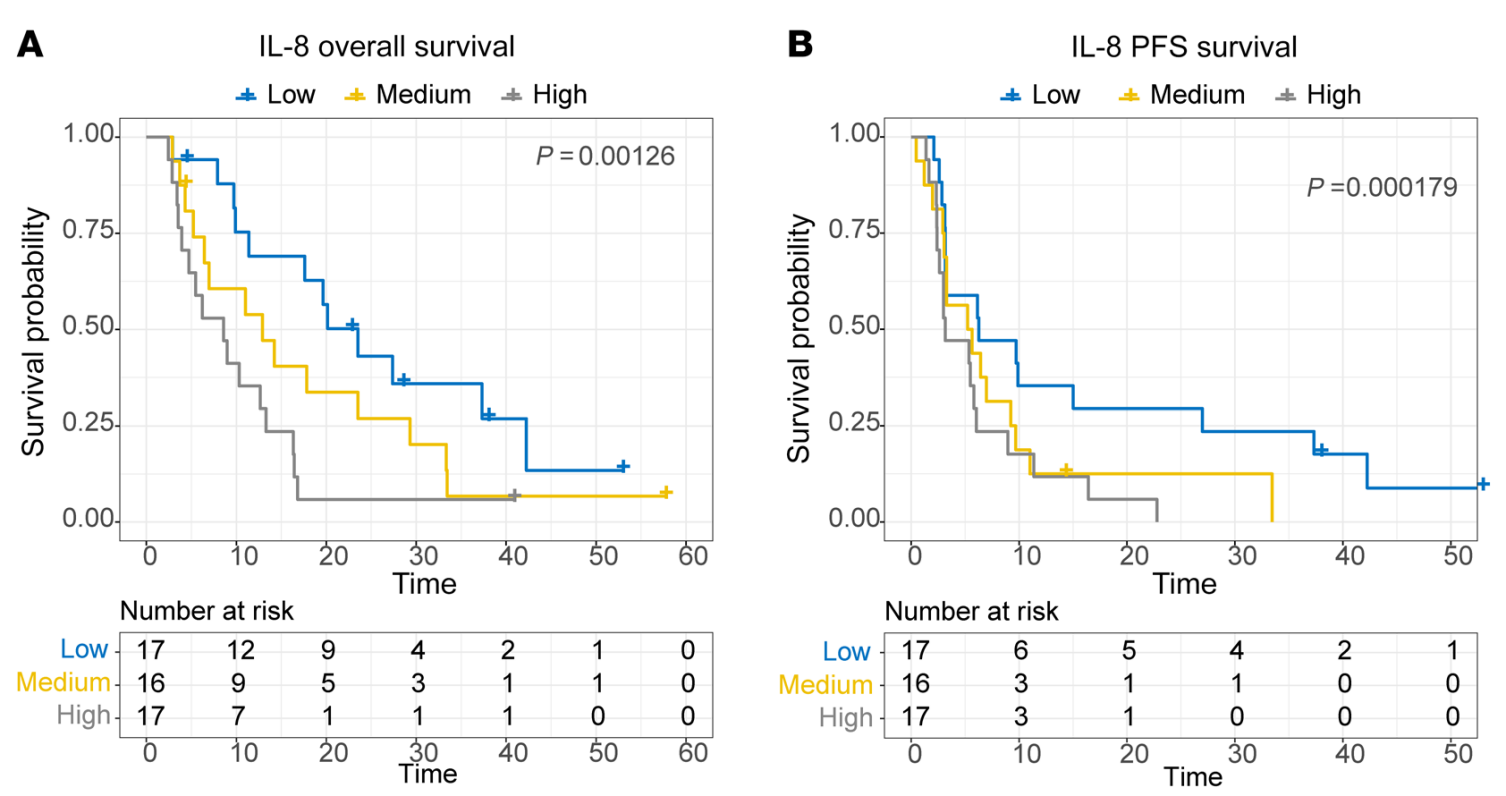
Increased serum IL-8 concentration is associated with reduced OS. Patient IL-8 cytokine concentration was split into tertiles (low, medium, and high concentration). Kaplan-Meier plots of association of IL-8 with (**A**) OS and (**B**) PFS across time (weeks). Significance was tested with Cox PH and log-rank *P* value is displayed on graph.

We analyzed the expression of 19 cytokines from pretreatment samples correlating low, medium, and high serum levels with clinical endpoints. Consistent with our previous study (26), we found that higher IL-8 correlated with both shorter OS, in univariable (*P* = 0.03) and multivariable (*P* = 0.001) analysis, and shorter PFS (*P* < 0.001) using Cox PH models (Figure 5). No other cytokines were significant in multivariable testing with PFS and OS (Supplemental Table 2). No significant relationship was found between IL-8 and the previously described pathway expression signatures identified in gene transcriptional analysis (19).

### RS correlates with response group.

**Figure 6:**
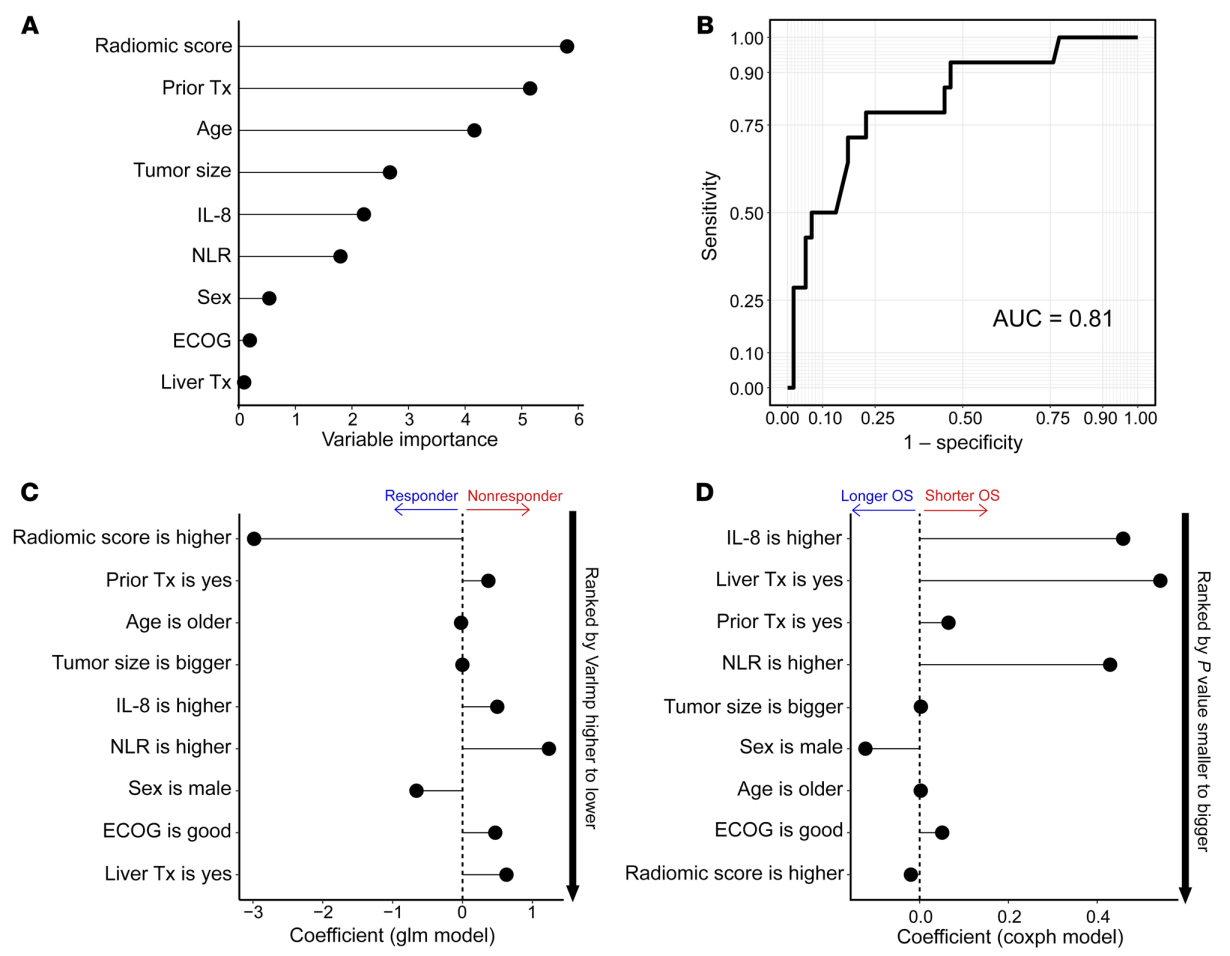
Multivariable modeling of response and OS correlation. (**A**) Variable importance (VarImp) of demographic, molecular, and relevant clinical features in distinguishing responders (R) and nonresponders (NR). (**B**) RF machine learning models for R/NR classification with 5-fold cross-validation. All 9 features were included. (**C**) Directionality of association between R/NR and each feature from **A** estimated by logistic regression model. Features were ranked by VarImp higher to lower in the same order as shown in **A**. (**D**) Association between features and probability of OS using multivariable Cox PH regression model. Features are ranked by *P* value (Wald’s test) from smaller to bigger. *n* = 72 patients (14R, 58NR) were used for analysis in **A**–**C**, *n* = 75 patients in **D**. Tx, treatment; glm, generalized linear model.

Among the 9 features in the random forest (RF) models, RS was the most important predictor of response, followed by any prior systemic treatment, age, tumor size, IL-8 cytokine levels, and neutrophil-to-lymphocyte ratio (NLR) (Figure 6A), with an overall performance of AUC 0.81 (Figure 6B). To determine the directionality of association between response and each feature, we additionally built a multivariable logistic regression model (Figure 6C). Three features were associated with response (higher RS, older age, and male gender), with the others associated with nonresponse. When stratifying by unirradiated and irradiated lesions (Supplemental Figure 1) the results were similar, with RS being a strong predictor of response, except the directionality of association between responders and nonresponders differed for unirradiated and irradiated lesions for NLR, IL-8 cytokine levels, and male sex. Furthermore, we sought to examine the association between each feature and survival outcome using a multivariable Cox PH regression model (Figure 6D). After ranking features from small to large by *P* values, we found that IL-8 cytokine level was the strongest predictor of OS, with increased IL-8 associated with higher risk of death. These results are consistent with our findings above and from our earlier work (26).

## Discussion

We previously reported the safety and efficacy of SBRT+P in patients with advanced solid tumors in a phase I clinical trial (18), showing statistically similar irradiated LF in 18 patients receiving partial-Rx compared with 50 patients receiving complete-Rx treatment in the context of immunotherapy. Here, with the incorporation of an expansion cohort of 41 patients with large tumors, we further support the safety of this approach with an overall DLT rate of under 10% and no new safety signals. We observed a low LF rate that was not statistically significantly different when comparing tumor coverage by the radiation prescription dose. Furthermore, we reconfirm that irradiated tumor response to SBRT+P is independently associated with improved OS, that pretreatment RS is prognostic for clinical endpoints, and that elevated IL-8 is associated with inferior outcomes. Overall, the well-tolerated nature of partial-Rx suggests that multisite SBRT (for no more than 4 metastases) for large (more than 65 cc) lesions is safe.

We have provided hypothesis-generating data that clinical response in patients with advanced solid tumors may be obtained by irradiating a metastasis with only partial coverage by the prescription dose (partial-Rx) when radiotherapy is combined with immune-checkpoint blockade. On a dosimetric level, previous studies have established that the minimum SBRT monotherapy dose required for local control of advanced solid tumors is 36 Gy in 3 fractions (19, 27, 28). In our study, all tumors had predefined SBRT prescription doses based on anatomical site (Table 3). Based on the minimum doses delivered to tumors in the partial-Rx treatments, significantly worse LF would be expected; although, the median D90% for the partial-Rx tumor’s PTV was 82%, which equates to a dose of 36.9 Gy in 3 fractions (above the minimum SBRT dose required for local control). The median D100% for the partial-Rx tumor’s PTV remains significantly lower than the minimum 36 Gy in 3 fractions. Despite the significantly lower D100% and D90% for the partial-Rx tumor’s PTV compared with complete-Rx tumor’s PTV(*P* < 0.01), there was no statistically significant difference in LF. Comparing tumors with LF to tumors without LF, there was no significant difference in the PTV D100% and D90% (*P* = 0.54). While the RS was significantly different comparing tumors that responded with tumors that progressed (*P* = 0.02), there was no significant difference in RS between partial-Rx and complete-Rx tumors (*P* = 0.35).

Additional within-patient comparisons showed that the majority (84%) of patients demonstrated convergent responses, meaning partial-Rx and complete-Rx tumors responded similarly within the same patient. Although Figure 1 shows that the LF for partial-Rx tumors is numerically higher than that for complete-Rx tumors and competing risks regression trend toward worse LF for partial-Rx versus complete-Rx tumors (HR 2.32, 95% CI 0.90–5.97, *P* = 0.08), there are other observable and nonobservable factors to consider. All partial-Rx tumors were more than 65 cc in volume, and larger tumors tend to have worse LF (29, 30). In addition, liver tumors tend to have worse LF (31), and the partial-Rx tumor cohort was enriched with large liver metastases, as evidenced by the fact that 36% of partial-Rx tumors were liver tumors, compared with 18% of complete-Rx tumors (Table 3). When controlling for anatomical site on a competing-risk regression, there was no longer a trend toward worse LF for partial-Rx tumors compared with complete-Rx tumors (HR 1.50, 95% CI 0.57–3.92, *P* = 0.41).

Despite the data suggesting similar LF for partial-Rx and complete-Rx tumors, this study was not designed to establish noninferiority of partial-Rx and complete-Rx treatments. Randomized data is needed to establish noninferior LF of partial-Rx and complete-Rx treatments in the context of immunotherapy. For example, a clinical trial could be conducted randomizing patients with metastatic, advanced, solid tumors receiving immunotherapy and SBRT versus no SBRT followed by a second randomization for the patients receiving SBRT as partial-Rx approach versus complete-Rx treatment. Ideally, the trial would be restricted to 1 or only a few primary histologies. This hypothetical trial could establish both the efficacy of adding SBRT to immunotherapy and of partial-Rx treatment in the setting of immunotherapy.

Proposed mechanisms of local response in large tumors observed with the use of SBRT+P, irrespective of PD-L1 status, include a local immune response to radiation. Several groups have found that SBRT is able to induce both innate and adaptive immune pathways, while downregulating genes involved in cell cycle and DNA damage repair pathways (32–34). It is well established that tumors with sustained responses to immunotherapy are associated with a T cell–inflamed tumor microenvironment characterized by tumor infiltrating lymphocytes (TILs) and type I/II IFN gene expression profiles (35, 36), while non-T cell–inflamed tumors are associated with decreased response to immunotherapy. This migration of TILs may be further augmented with the synergistic use of radiation and checkpoint inhibitor therapy (37). Moreover, intratumoral T cells exposed to radiation have shown the ability to survive, undergo reprogramming, increase motility within the tumor microenvironment, and produce higher levels of IFN-γ, thus stimulating a more robust immune infiltrate (38). Specifically in this patient population, we previously demonstrated from biopsies of irradiated tumors that increased expression of intratumoral cytolytic T cell genes after SBRT was associated with an improved tumor response (19).

To our knowledge, this is the first study to report that the magnitude of irradiated tumor response to SBRT+P is associated with OS, with responders to SBRT+P demonstrating improved OS compared with nonresponders. These findings were independently supported on multivariable analysis when controlling for other clinical factors. Two hypotheses for this improvement in OS might be cytoreduction in symptomatic tumor burden and/or T cell reinvigoration (39). Based on these data, multi-site SBRT can cytoreduce large, symptomatic, tumors while also potentially augmenting antitumor immune responses to improve survival when combined with immune-checkpoint blockade.

Our findings provide a rationale to prioritize sparing radiation dose to adjacent OARs while providing maximum tumor dose (1, 40) as was permitted on the oligometastatic trials NRG BR001 (41) and BR002 (15). Delivering a high dose to most of a metastasis while protecting normal organs may be a strategy to expand patients eligible for oligometastatic (42, 43), oligoprogressive (44), and more recently the ARREST (Ablative Radiation Therapy to Restrain Everything Safely Treatable) (45) treatment paradigms. Other areas where large tumor partial-Rx SBRT might be relevant could also include reirradiation, dose escalation (40), and nonoperative, large, primary tumors with nearby critical OARs (46).

The development of noninvasive biomarkers of treatment response and resistance is an unmet need in oncology and here we propose both the RS and circulating IL-8 levels as potential predictors of outcome. The ability to identify tumors with a T cell–inflamed microenvironment using the RS may allow for improved patient outcomes, treatment modality, and, potentially, even tumor-specific treatment selection for SBRT. IL-8 (CXCL8), which binds to CXCR1/2 receptors promoting neutrophil recruitment and activation (47), is a potential biomarker for RT and immunotherapy resistance (26, 48, 49). The protumor and immunosuppressive functions of IL-8 have been described via several mechanisms, including, but not limited to, myeloid-derived suppressor cells and neutrophil extracellular traps, angiogenesis and metastasis, epithelial-mesenchymal transition, and renewal of cancer stem cells (50–55). In this study, we performed a multi-variable correlation of IL-8, observing association with inferior outcomes but no association with tumor size, lines of therapy, or RS. This would suggest an independent effect of IL-8 and raise the possibility of jointly targeting IL-8 with SBRT and anti-PD1. The combination of BMS-986253 (a humanized IgG1 anti-IL-8 monoclonal antibody) and nivolumab has demonstrated safety and preliminary efficacy in patients with melanoma who have progressed on earlier anti-PD1 therapy (56), and has been shown to result in suppression of circulating IL-8 as well as intra-tumoral CD15^+^ neutrophils (57). Leveraging our observations surrounding IL-8 and these clinical trial data, we have launched a phase I study of SBRT with nivolumab and BMS-986253 (NCT04572451) (58).

There are limitations to our current investigation. Several patients were excluded from the analysis because they either died before receiving SBRT+P or died before a radiologic assessment, rendering them nonevaluable. The exclusion of these patients may lead to bias, although the landmark analysis was intended to account for this. To address the high mortality rate, we used competing-risks regression to account for the fact that many patients died before developing a LF. There are many plausible explanations for the lack of an observed significant difference in LF between partial-Rx and complete-Rx tumors, one of which being a synergistic relationship between pembrolizumab and SBRT, however, an additive relationship is also possible. The heterogeneous nature of the cohort with various tumor histologies, locations, and volumes, as well as overall PD-L1 data, may introduce bias. We are not able to make claims for a specific tumor type, but future studies are warranted for individual tumor types and perhaps as an earlier line of therapy. The RS is dependent on imaging parameters (59) such as scanner type, reconstruction approach and user dependency on target delineation. Our RF machine learning model will also need to be validated in an independent cohort.

In conclusion, these findings demonstrate the safety and feasibility of SBRT and PD-1 immunotherapy in patients with large, advanced, solid tumors. We observe that partial-Rx has comparable LF to complete-Rx in our cohort of patients with large, advanced, solid tumors. Moreover, posttreatment tumor response to SBRT+P is associated with survival, and pretreatment RSs as well as elevated IL-8 deserve further study as predictive biomarkers. A randomized trial is warranted utilizing multi-site SBRT, including partial tumor irradiation, in the setting of immune-checkpoint blockade.

## Methods

### Study design.

In the original publication of this phase I clinical trial (18), the primary objective was to determine the recommended SBRT dose to different anatomic locations before pembrolizumab. Secondary objectives included irradiated LF (stratified by coverage of tumor by radiation prescription dose), grade 3+ adverse events, response rate, PFS, OS, immune score gene–expression analysis, and changes in the tumor microenvironment induced by radiation. This study was not specifically designed to establish noninferiority of treating the entire tumor versus part of the tumor with the radiation prescription dose. Decreased radiation doses were predefined and expected to be used if the cumulative dose-limiting toxicity rate at 3 months was greater than or equal to 33% in any anatomical cohort. The radiation doses and anatomical cohorts are shown in Table 3 as well as the number of complete-Rx and partial-Rx tumors. The reduced radiation doses were never used due to the low DLT rate.

### Patients.

Patients with an Eastern Cooperative Oncology Group (ECOG) performance status of at least 1 were eligible for enrollment if they were at least 18 years old with a metastatic solid tumor previously treated with standard-of-care therapy. Measurable disease by RECIST, version 1.1, was required with at least 2 tumors. In the initial dose–determination cohort, tumors ranging from 0.25–65 cc that were amenable to SBRT were allowed, and tumors measuring more than 65 cc were treated with partial irradiation. Patients in this cohort were enrolled between January 12, 2016 and March 3, 2017. An expansion cohort of patients with at least 1 tumor of more than 5 cm (more than 65 cc) was later opened to further explore SBRT+P in this advanced, solid tumor patient population. In the expansion cohort, patients were enrolled between December 27, 2017 and November 20, 2019. Data lock occurred on May 16, 2021.

### Radiation technique.

Patients received SBRT to at least 2 measurable metastases with each lesion receiving 30–50 Gy over 3-5 fractions depending on anatomical location (18, 60). For multisite SBRT, consensus was established to limit the target metastasis size to a 65 cc volume and the number of metastases targeted in a patient to 4 (18). Due to logistical constraints of SBRT planning, not all metastases were targeted. Metastases were prioritized based on the largest size and/or those causing the most morbidity. For tumors over 65 cc, a target volume was created within the gross tumor volume to limit the treated area of the tumor to a maximum of 65 cc (partial-Rx). To create this 65 cc volume, a uniform volumetric contraction was initiated from the edge of the gross tumor volume/internal target volume (GTV/ITV) until a 65 cc volume was reached. This standardized uniform contraction internal target volume (SUCITV) was then expanded by 0.5 cm to create the PTV. For GTVs/ITVs of less than or equal to 65 cc, these volumes underwent a similar 0.5 cm PTV expansion and were covered completely by the prescription dose (complete-Rx), respecting adjacent OAR tolerances.

For nonthoracic or abdominal tumors away from the diaphragm, the GTVs were delineated on axial slices from the CT simulation. For thoracic or abdominal tumors closer to the diaphragm with tumors that moved no more than 1cm on 4 DCT, an ITV was generated with the aid of maximum-intensity projection (thoracic) or minimum-intensity projection (abdomen) images. For those that moved more than 1cm on 4 DCT, either a breath-hold approach was utilized to generate a GTV or abdominal compression devices were used to generate an ITV at the discretion of the radiation oncologist. SBRT dose varied by anatomical site: 45 Gy in 3 fractions for peripheral lung, liver, and abdominal/pelvic; 50 Gy in 5 fractions for central lung and mediastinal/cervical/axillary; 30 Gy in 3 fractions for osseous and spinal/paraspinal. If excess toxicity was observed, a reduced dose was specified. Treatment was delivered using photons with linear accelerators once daily on an every-other-day basis within 14 days. Pembrolizumab (200 mg IV every 3 weeks) began within 7 days following the last fraction of SBRT (SBRT+P) and was continued for a maximum of 2 years or until radiographic or clinical progression.

### Follow-up.

Tumor assessments were performed every 2–3 months utilizing RECIST guidelines modified to allow irradiation of target lesions. Osseous and spinal metastases were considered controlled/progressed as part of the LF measurement as per MD Anderson response criteria (61). Under FDA guidance, DLTs were attributed to the combination of SBRT+P therapy by anatomical organ system, and not to each individual therapy.

### RSs.

RSs were calculated using previously reported methods from pretreatment CT simulation images acquired from a Philips CT scanner (20). CT images were obtained with or without iodinated contrast, as appropriate, had a slice thickness of no more than 3 mm, and used conventional convolution kernel reconstruction. Tumors were then delineated using Pinnacle (Philips Radiation Oncology Systems) or Eclipse (Varian Medical Systems). A separate ring structure, approximating the tumor microenvironment, was created by performing a 2 mm isotropic expansion and contraction on the original tumor boundary. All radiomic feature extraction was done using the LIFEx software (version 4.60) (62). Images were resampled to 1 × 1 × 1 mm^3^ voxels within the software by use of 3-dimensional Lagrangian polygon interpolation. Hounsfield units in all images were resampled into 400 discrete values ranging from –1,000 to 3,000. The RS was then calculated using 8 variables, which included 5 radiomics features, 2 volume-of-interest locations, and the peak kilovoltage used on the CT scanner.

RS was calculated for each irradiated tumor (metastasis-level) and for each patient (patient-level). The patient-level RS was generated by averaging the RSs from all irradiated metastases within each patient. First, the RS was analyzed as a continuous variable at both the metastasis and patient levels. Second, the RS was dichotomized into 2 groups using the previously reported low- and high-RS cutoff values in patients with large, advanced, solid tumors (22). Since the RS is correlated with the degree of CD8^+^ T cell infiltration, the low-RS group corresponded to tumors (or patients with tumors) with low CD8^+^ T cell infiltration and the high-RS group corresponded to tumors (or patients with tumors) with high CD8^+^ T cell infiltration.

### Cytokine analysis.

Pretreatment serum cytokine levels were detected using the V-PLEX Proinflammatory Panel 1 Human Kit (Meso Scale Discovery) and the V-PLEX Cytokine Panel 1 Human Kit (Meso Scale Discovery) per manufacturer instructions. Samples were read on MSD Quickplex SQ120 instrument and analyzed using the MSD Discovery Workbench Data Analysis Software. Concentrations for each cytokine were log_2_ transformed and split into tertiles, which were categorized into low, medium, or high expression groups. For each cytokine: (a) association of RS with OS, PFS, and LF was tested using Cox PH models, (b) association of RS with continuous variables including tumor diameter, GTV (tumor volume), and treatment response (unirradiated lesion response, irradiated lesion response and overall RECIST) was tested using linear regression models, (c) association of RS with categorical variables was tested using logistic regression models. Multinomial logistic regression (uni- and multi-variable) of response was performed at last scan comparing progressive disease versus partial response (PD versus PR) or progressive disease plus stable disease versus partial response (PD+SD versus PR). Covariates included age, sex, smoking history, ECOG performance status, baseline neutrophil/lymphocyte ratio, number of prior systemic therapy regimens, PD-L1, and presence of liver metastases. The Benjamini-Hochberg (BH) FDR method was used for adjusting multiple comparisons.

RF classification models were used to estimate the variable importance of demographic, molecular, and relevant clinical features for prediction of response group. Response group was defined as Overall RECIST Binary ORR (meaning at least 30% tumor shrinkage of target lesions was R, otherwise NR). A total of 75 patients had both cytokine and clinical data. After removing 1 patient with missing response and 2 patients with missing RSs, 72 patients (14R, 58NR) with complete data were included. Considering the limited number of responding patients available for model optimization, we chose not to split the data set into training and test sets. Rather, we leveraged data from all patients to build as robust a model as possible using models trained with 5-fold cross-validation. 9 features were included: (a) demographic: age (in years), sex (female/male); (b) clinical: liver treatment (yes/no), prior systemic treatment (yes/no), ECOG performance status (0[fully active, denoted as good]/1[not fully active, denoted as bad]), tumor size (MTD[maximum transverse diameter] in mm); (c) molecular: NLR, IL-8 cytokine level, RSs. Categorical variables were converted to dummy variables using R function *dummyVars* with parameter fullRank set to TRUE. Data were preprocessed to remove features that have near-zero variance, high correlation (Spearman’s *P* > 0.75), or high collinearity. All features passed QC and were kept for analysis. Each feature was scaled and centered. Logistic regression models were additionally used to determine directionality of association between response group and each feature, with negative and positive coefficients indicating enrichment or higher value in R or NR, respectively. R packages *caret* (v6.0–90) and R function *glm* (v4.1.1) were used.

Cox PH regression models were used to estimate the association between OS and demographic, molecular, and relevant clinical features. Patients who were alive or lost contact before end of last follow up date were censored (right censoring). The same 9 features listed above were included in the model. Seventy-five patients were used for analysis, as there was no need to remove patients with missing data for Cox models. Negative and positive association between probability of survival and each feature was indicated by coefficients of the model, respectively. Those coefficients (coef) can be directly converted to HR by *HR* = *e^coef^* (e as Euler’s number). *P* values were computed by Wald’s test. R packages survival (v3.2.11) were used.

### Statistics.

A combined analysis of individual patient data from both the initial dose–determination cohort and expansion cohorts was performed. Baseline characteristics and radiation dosimetry were compared between patients in the complete-Rx cohort (all treated tumors received complete coverage by the prescription dose) and the partial-Rx cohort (patients with at least 1 tumor receiving partial coverage by the prescription dose) using *t* tests or Wilcoxon rank-sum tests and Pearson’s χ^2^ or Fisher’s Exact tests for continuous and discrete variables, respectively. Patients were considered analyzable if they received the protocol treatment (SBRT and at least 1 cycle of Pembrolizumab), and if they were evaluable for toxicity and tumor control. Irradiated LF was defined as progression of an irradiated metastasis. The association of LF and select covariates was assessed using *t* tests or Wilcoxon rank-sum tests and Pearson’s χ^2^ or Fisher’s Exact tests for continuous and discrete variables, respectively. LF was analyzed using the cumulative incidence estimator to accurately estimate the cumulative probability of LF in the presence of competing death events. This method was deemed more appropriate for this cohort compared with the Kaplan-Meier method, which censors at the time of death for patients who died without irradiated tumor progression (63). At the first scan, irradiated metastasis response was defined as follows: patients who had a 30% or greater decrease (50% for osseous/spine) in the maximum transverse diameter of each irradiated metastasis were classified as responders, and those who had a 20% or greater growth (25% for osseous/spine) in the maximum transverse diameter of 1 or more irradiated metastases were nonresponders. Patients who did not fit either category were deemed to have stable disease (mixed-responders). Irradiated metastasis response was evaluated using shared frailty Cox regression models and generalized estimating equations to account for correlation of individual metastasis responses within patients. We used the Kaplan-Meier method to estimate PFS, utilizing the time from SBRT to either progression or death from any cause, and OS, using the time from SBRT to death. Landmark analysis at 2 months was used to avoid a guarantee-time bias when examining the association of irradiated metastasis response with OS (64). The RS was examined as both a continuous variable and as a dichotomous variable using the previously reported low- and high-RS cutoff values (22) to evaluate its association with LF, PFS, and OS. We stratified outcomes using clinical variables and RS, and then compared differences using the log-rank test and Cox regression modeling. Baseline PD-L1 staining was available from 70 patients, and a cut-off of at least 1% was used to define PD-L1 positivity. PD-L1 scoring was done by tumor proportion score (TPS). *P* < 0.05 was considered statistically significant. Multivariable Cox regression analysis was performed for covariates with univariable *P* < 0.10 or those with historical significance. Data were analyzed using Stata, version 16.0 (StataCorp).

### Study approval.

Study enrollment for both cohorts began after the IRB approved the study; written informed consent was obtained and pursued per Declaration of Helsinki (clinicaltrials.gov: NCT02608385) (18).

## Author contributions

MCK and BEO took part in writing and analysis. The order of first authorship was determined because there was equal contribution to writing and presentation, but more analysis from MCK. RED was responsible for writing and analysis. JHH, LK, and ACO were responsible for writing. YZ conducted experiments and acquired data. TGK was responsible for analysis and review of the final manuscript. GFF was responsible for design, review of final manuscript, and acquiring data. RRW participated in project design. RB was responsible for writing, design, and analysis. SJC was responsible for design and investigation. JJL was responsible for design, investigation, analysis, and writing.

## Supplementary Material

Supplemental data

ICMJE disclosure forms

## Figures and Tables

**Table 2:**
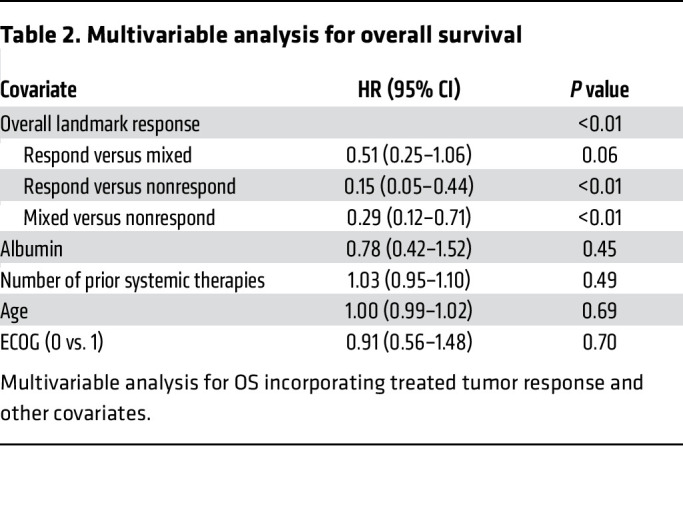
Multivariable analysis for overall survival

**Table 1:**
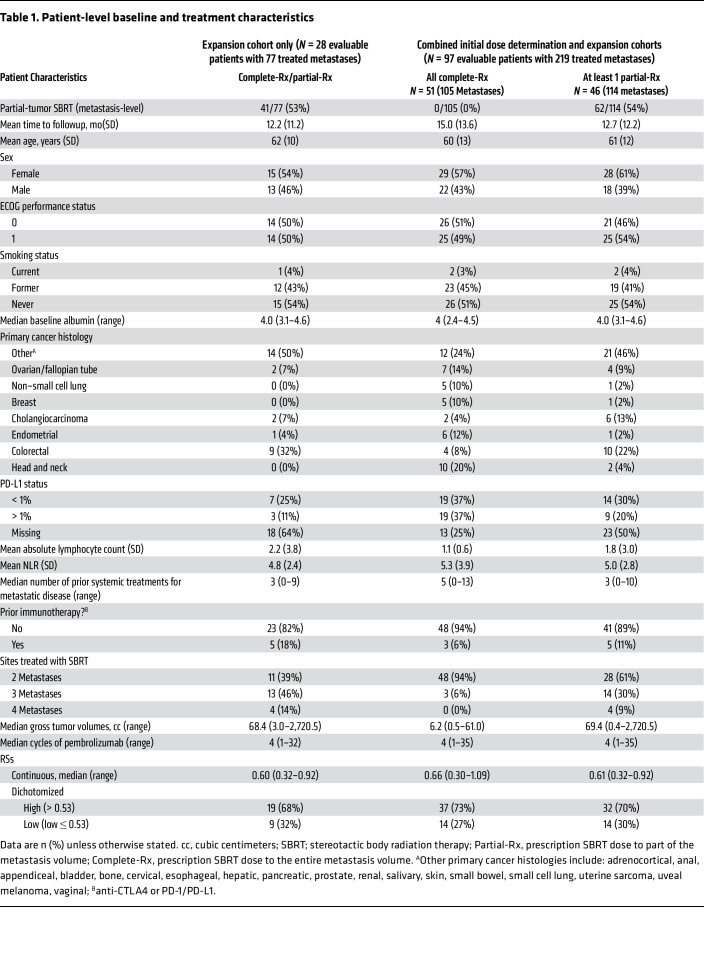
Patient-level baseline and treatment characteristics

**Table 3:**
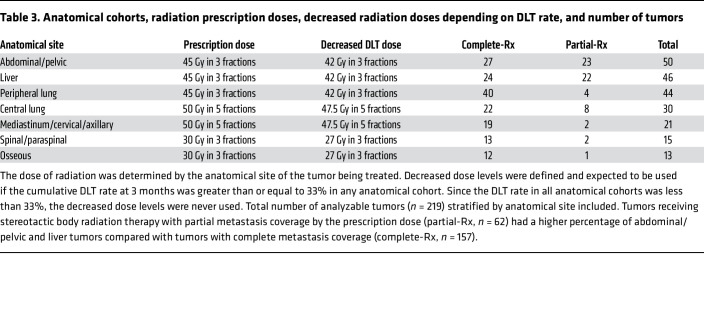
Anatomical cohorts, radiation prescription doses, decreased radiation doses depending on DLT rate, and number of tumors

## Version 1 05/15/2023

Electronic publication
