# Supplementary material for: Partial tumor irradiation plus pembrolizumab in treating large advanced solid tumor metastases
Source: J Clin Invest. 2023 May 15;133(10):e162260. doi: 10.1172/JCI162260 (PMC10178837; doi:10.1172/JCI162260)
Supplement: Supplemental data [file jci-133-162260-s137.pdf]

Supplementary Figures

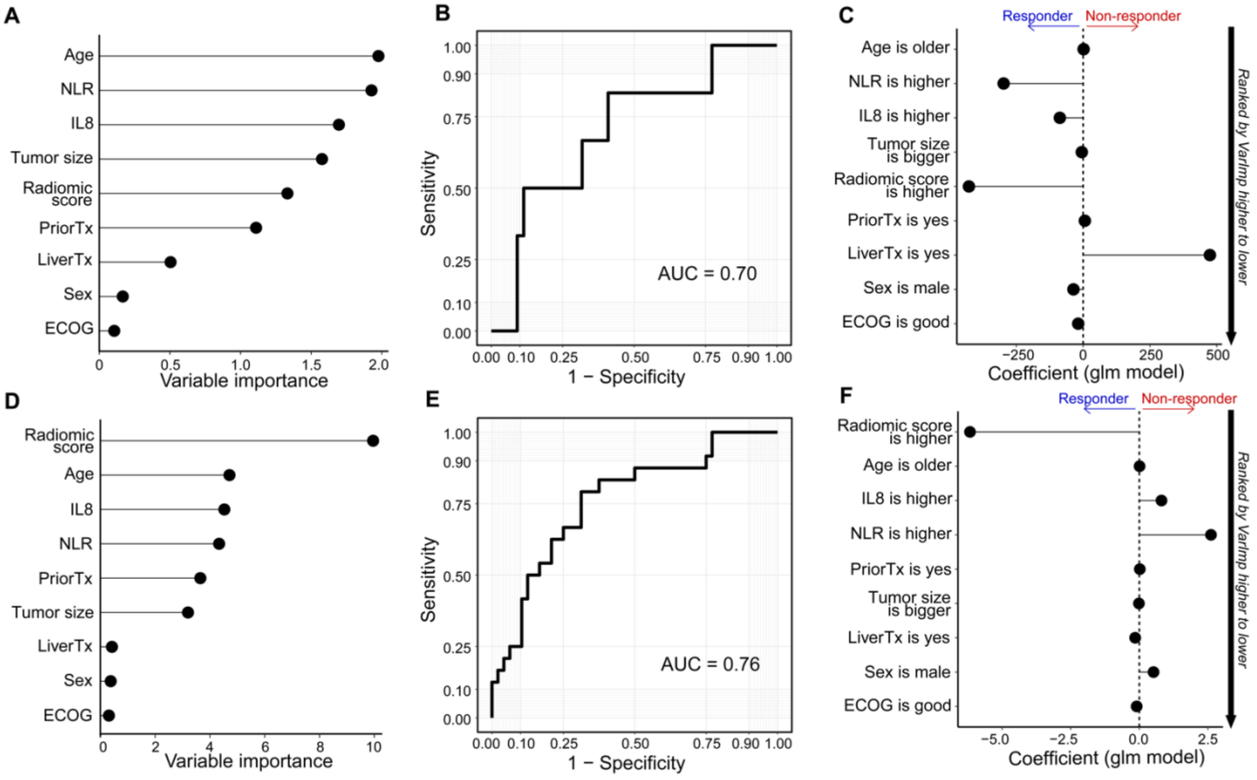

Figure S1. Multivariable modeling of response stratified by unirradiated (A-C) and irradiated lesions (D-F). **(A)** Variable importance (VarImp) of demographic, molecular, and relevant clinical features in distinguishing responders (R) and non-responders (NR) for unirradiated lesions only. **(B)** Random Forests (RF) machine learning models for R/NR classification with 5-fold cross-validation for unirradiated lesions only. All nine features were included. **(C)** Directionality of association between R/NR and each feature from (A) estimated by logistic regression model for unirradiated lesions only. Features were ranked by VarImp higher to lower in the same order as shown in (A). **(D)** VarImp of demographic, molecular, and relevant clinical features in distinguishing R and NR for irradiated lesions only. **(E)** RF machine learning models for R/NR classification with 5-fold cross-validation for irradiated lesions only.

All nine features were included. **(F)** Directionality of association between R/NR and each feature from (D) estimated by logistic regression model for irradiated lesions only. Features were ranked by VarImp higher to lower in the same order as shown in (D). *n*=50 patients were used for analysis in A-C. *n*=72 patients were used for analysis in D-F. Patients with missing data or response unavailable were excluded from analysis. AUC, area under curve; Tx, treatment; glm, generalized linear model.

Supplementary Tables

Covariate	P Value
Anatomic Site	<0.01
Radiomics Score (continuous)	0.02
Partial-Rx vs Complete-Rx	0.09
PD-L1 (%)	0.56
Histology	0.18
Age	0.42
ECOG (0 vs 1)	0.45
Number of Prior Systemic Therapies	0.62
Prior Immunotherapy	0.38
Tumor Volume (cc)	0.68

Table S1: Association of local failure and select covariates. Association between local failure and select covariates was evaluated using t-tests or Wilcoxon rank-sum tests and Pearson's χ^2 or Fisher's Exact tests for continuous and discrete variables, respectively. Only anatomic site and the radiomics score were found to be associated with local failure.

Cytokine	UVA p-value (OS)	UVA p-value (PFS)	MVA p-value (OS)	MVA p-value (PFS)
GM-CSF	0.73	0.71	0.40	0.35
IFNγ	0.73	0.82	0.20	0.89
IL-10	0.74	0.80	0.69	0.69
IL-12.IL-23p40	0.74	0.62	0.25	0.23
IL-12p70	0.41	0.97	0.15	0.20
IL-13	0.73	0.71	0.33	0.88
IL-15	0.86	0.80	0.40	0.67
IL-16	0.77	0.71	0.15	0.21
IL-17A	0.74	0.80	0.15	0.20
IL-1α	0.41	0.71	N/A	N/A
IL-2	0.41	0.71	0.15	0.20
IL-4	0.05	0.03	0.09	0.02
IL-5	0.74	0.80	0.51	0.29
IL-6	0.74	0.80	0.15	0.21
IL-7	0.86	0.80	0.25	0.36
IL-8	0.03	0.37	<0.01	<0.01
TNF-α	0.41	0.80	0.21	0.69
TNF-β	0.74	0.80	0.06	0.20
VEGF	0.74	0.80	0.09	0.04

Table S2. Pre-treatment serum cytokine correlation with overall survival (OS) and progression-free survival (PFS). IL-8 was the only cytokine to demonstrate a statistically significant correlation with both overall survival (OS) and progression-free survival (PFS) on univariable (UVA) and multivariable (MVA) testing.
